# Complete genome sequence of *Pseudomonas* sp. SCT, an iodate-reducing bacterium isolated from marine sediment in Japan

**DOI:** 10.1128/mra.00974-25

**Published:** 2025-11-26

**Authors:** Haruna Kuge, Haruhiko Sato, Shigeki Yamamura, Seigo Amachi

**Affiliations:** 1 Graduate School of Horticulture, Chiba University 12737, Matsudo, Japan; 2 Regional Environment Conservation Division, National Institute for Environmental Studies 13585, Tsukuba, Japan; Montana State University, Bozeman, Montana, USA

**Keywords:** *Pseudomonas*, complete genome

## Abstract

*Pseudomonas* sp. strain SCT is a dissimilatory iodate (IO_3_^–^)-reducing bacterium isolated from marine sediment in Japan. Here, we report the complete genome sequence of strain SCT.

## ANNOUNCEMENT

A novel dissimilatory iodate-reducing bacterium *Pseudomonas* sp. SCT was isolated from marine sediment, which was collected from the upper 3 cm of Sagami Bay, Kanagawa, Japan (35°N, 139°E) on 1 April 2004 (1). The strain was isolated by serial dilution and a single-colony isolation technique after several enrichments with acetate and iodate as the electron donor and acceptor, respectively. Phylogenetic analysis based on 16S rRNA gene revealed that strain SCT was closely related to *Pseudomonas stutzeri*. In 2019, we reported the draft genome sequence of this strain (2). However, the draft genome is insufficient for detailed analysis of this unique bacterium, such as the identification of complete genes necessary for dissimilatory iodate reduction. Here, we report the complete genome sequence of strain SCT, which provides a basis for future functional and comparative genomic analysis.

Strain SCT was grown anaerobically for 3 days at 30°C in a minimum medium (1) containing acetate (10 mM) and iodate (4 mM). The genomic DNA was extracted using a DNeasy Blood and Tissue Kit (Qiagen, Hilden, Germany) according to the manufacturer’s instructions. High-molecular weight DNA was sheared using the Megaruptor 3 (Diagenode), and fragment size distribution was assessed with the Femto Pulse System (Agilent). Size selection was performed using AMPure PB magnetic beads (Pacific Bioscience). A total of 10 µL of SMRTbell library was prepared with the PacBio SMRTbell prep kit 3.0 according to the manufacturer’s instructions. The SMRTbell templates were then annealed and bound using the Sequel II Binding Kit 3.2 together with Int Ctrl 3.2. Sequencing was conducted on the PacBio Sequel II system, and circular consensus sequences (CCS; HiFi reads) were generated using SMRT Link v11.1.0 with automatic adapter removal and *Q* ≥ 35 filtering, yielding 0.2 Gb of HiFi reads (3). The mean number of passes per molecule was 12. No additional trimming or polishing was performed. The HiFi reads had an *N*_50_ of 10,659 bp, and a total of 106,482 reads were obtained. Genome assembly was carried out using the Microbial Genome Assembly pipeline in SMRT Link v11.1.0 and, in parallel, with Flye v2.4.2 using default parameters. Two contigs were obtained: contig1 (4,789,185 bp; G + C content, 62.5%) and contig2 (30,796 bp; G + C content, 61.8%) (Table 1). Assembly completeness was assessed using BUSCO v5.1.3 (4) with the bacteria_odb10 data set, identifying 124 BUSCO groups, all of which were classified as complete (100%).

**TABLE 1:** Overview of data sets

	Size (bp)	GC (%)	Depth	Circular
Contig 1	4,789,185	62.5	213.6	Yes
Contig 2	30,796	61.8	238.2	Yes
Total	4,819,981	62.5	213.8	

After genome assembly, sequence homology was assessed by database searches using BLAST + v2.7.1+ (5). Gene prediction and annotation were performed with the DDBJ Fast Annotation and Submission Tool (DFAST) v.1.6.0 (6). Contig 1 contained 4,505 coding sequences, 12 ribosomal RNA (rRNA) genes, and 61 transfer RNA (tRNA) genes, whereas contig 2 contained 34 coding sequences and no rRNAs or tRNA genes.

## Data Availability

The BioProject accession number is PRJDB18120. The GenBank accession numbers are AP031612 (contig 1), AP031613 (contig 2), and GCA_041215865 (total). The BioSample accession number is SAMD00786919. The PacBio raw data accession number is DRR555124.
